# Amide-Assisted Rearrangement of Hydroxyarylformimidoyl Chloride to Diarylurea

**DOI:** 10.3390/molecules26216437

**Published:** 2021-10-25

**Authors:** Xizhong Song, Xiaoyu Liu, Wei Yu, Yi Jin

**Affiliations:** 1 Key Laboratory of Medicinal Chemistry for Natural Resource, Ministry of Education, Yunnan Provincial Center for Research & Development of Natural Products, School of Chemical Science and Technology, Yunnan University, Kunming 650091, China; song519672429@126.com (X.S.); eachnet99@126.com (X.L.); 2 Jianxi Nafutang Pharmaceutical Co., Ltd., Zhangshu 331200, China; 3 920th Hospital of Joint Logistics Support Force, PLA, Kunming 650118, China

**Keywords:** rearrangement, Diarylurea, benzamide, hydroxyarylformimidoyl chloride

## Abstract

A novel amide-assisted rearrangement reaction of hydroxybenzimidoyl chloride has been established for the efficient synthesis of 1,3-diphenylurea derivatives. A variety of electronically and sterically different 1,3-diphenylurea derivatives can be obtained in good to excellent yields, and a proposed reaction mechanism is also presented.

## 1. Introduction

**Figure 1:**
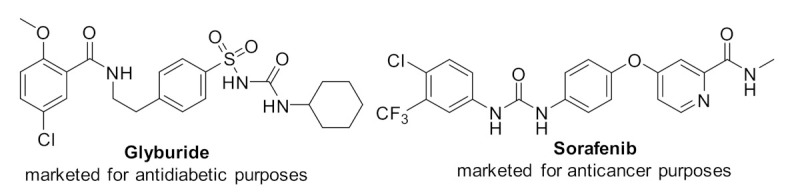
Representative biological urea derivatives.

Urea derivatives have a myriad of applications in biological studies, analytical chemistry, pharmaceuticals, polymer sciences, and agrochemicals [1,2,3,4,5,6,7,8]. N, N′-disubstituted urea exhibits a wide range of potent biological properties in bioactive and pharmacologically impressive structures [9,10,11,12,13,14]. For instance, many urea-containing compounds have been used to cure human diseases (Figure 1) [15,16,17,18].

**Scheme 1:**
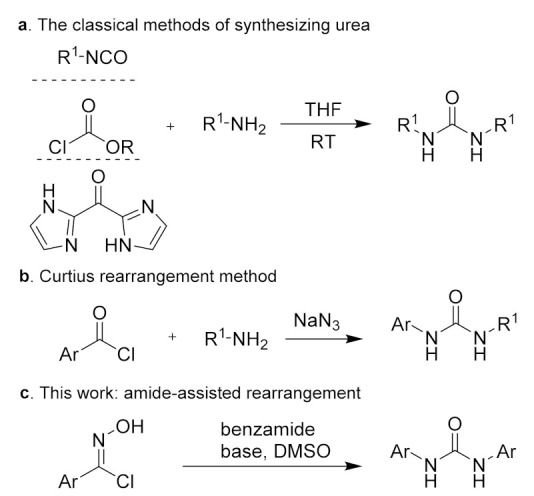
Preparation methods of N, N′-disubstituted urea.

Given the medicinal and biological properties of N, N′-disubstituted urea, synthetic–organic chemists and medicinal chemists have shown considerable interest in the development of efficient methodologies for the synthesis of this structure. The traditional methods of synthesizing urea involve the condensation reaction between an amine and active carbonyl compounds, such as isocyanate [19,20,21,22], chloroformate [23], and carbonyl di-imidazole [24,25] (Scheme 1a). Also, the Curtius rearrangement provides an effective method for preparing urea from an arylformyl chloride substrate (Scheme 1b) [26,27,28]. These methods have been extensively studied and applied in actual production, but the development of novel methods for the preparation of urea is still in high demand.

Aromatic oxime is a vital precursor and intermediate in organic synthesis [29,30,31]. The typical Beckman rearrangement reaction can achieve the conversion of ketoxime to amide products under strong acid conditions [32,33]. Since the hydrogen-atom migration of aldoxime is difficult, metal catalysts are usually required in order to carry out the Beckmann rearrangement of aldoxime [34,35,36,37]. Therefore, it is still challenging to achieve a metal-free aldoxime rearrangement. The amides have been widely used as directing groups to activate C‒H bonds and facilitate the conversion of multiple functional groups [38,39,40,41,42]. To date, there have been no reports of amide-assisted rearrangement reactions. We envision that the amide can utilize the hydrogen bond to bind with the hydroxyl group of hydroxyarylformimidoyl chloride, thereby activating the N‒O bond, which is conducive to the departure of the hydrated positive ion and allows the rearrangement of aldoxime.

Herein, we have developed a smooth and efficient synthesis of 1,3-diphenylurea derivatives from hydroxybenzimidoyl chloride under mild conditions with amides as additives.

## 2. Results and Discussion

In our initial studies, N-hydroxybenzimidoyl chloride (**1a**) was reacted with benzamide using N, N-dimethylformamide (DMF) as the solvent, and the desired product **2a** was obtained in medium yields (31%, Table 1, entry 1). Different solvents, such as methanol, ethanol, 1,4-dioxane, tetrahydrofuran (THF), dimethylsulfoxide (DMSO), and dichloromethane (DCM), were screened at room temperature. The results indicate that the most efficacious reaction occurred with DMSO (yield 42%, Table 1, entry 8). Encouraged by this result, we sought to enhance the yield of this reaction and carried out a screening of bases, such as K_2_CO_3_, Et_3_N, DMAP, DBU, and *t*-BuOK (Table 1, entries 10–14). The experimental data showed that the reaction proceeded with good yield (42%, Table 1, entry 8) when Cs_2_CO_3_ was used, while the other bases were not as effective. The reaction could not be carried out in the absence of a base (Table 1, entry 15). Additionally, increasing temperature is beneficial to the reaction, since higher yields were obtained at 120 °C (87%, Table 1, entry 21). Under these conditions, increasing the reaction time did not affect the yield (87%, Table 1, entry 23). Subsequently, we selected acetamide, propionamide, 2-phenylacetamide, and 4-chlorobenzamide to screen the additives. The results show that the effect of using benzamide is still more potent than the other amides. Simple amides, such as acetamide and propionamide, are also effective, but 2-phenylacetamide and 4-chlorobenzamide are not as effective (Table 1, entries 24–27). Notably, without the addition of the amide reagents, the reactions described herein will not occur (Table 1, entries 28). Therefore, under optimized conditions (using DMSO as the solvent with Cs_2_CO_3_ as the base at 120 ^°^C for 5 h), different N-hydroximoyl chlorides were selected in order to prepare products **2a**–**2l** (yield: 71–87%, Table 2). Reducing the amount of benzamide will significantly reduce the reaction yield (Table 1, entries 29–30), although the reaction yield did not increase significantly with an increase in the amount of benzamide, (Table 1, entries 31).

In Table 2, the results show that N-hydroxybenzimidoyl chloride (**1**) substrates that bore electron-donating groups (such as methoxy or methyl) as the R substituents were well-tolerated at good yields (**2e**, 83%, **2f**, 80%, **2h**, 84%, **2j**, 86%). In addition, electron-withdrawing substituents (such as chloro-, fluoro- or trifluoromethyl) are also usable in the reaction, but the reaction yield is reduced (**2c**, 72%, **2i**, 76%, **2k**, 71%, **2l**, 73%). Furthermore, the yield of *para*-containing substituents is higher than the yield of *meta*-containing substituents (**2c**, 72%, **2i**, 76%). Finally, the reaction yield of a substrate that contains two groups is not as high as a substrate that contains one group (**2b**, 77%, **2j**, 86%). Unfortunately, no corresponding products were obtained using other heteroaromatic substrates (**1**), such as pyridine, furane or thiophene.

**Figure 2:**
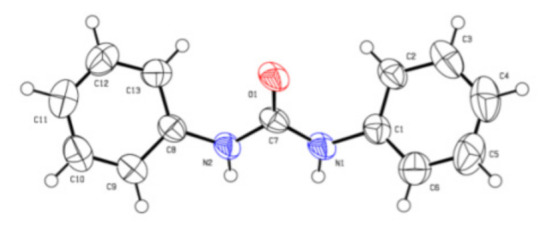
Crystal structure of **2a**.

The chemical structures of the 1,3-diphenylurea derivatives were examined by ^1^H NMR, ^13^C NMR, and HRMS analyses (see Supplementary Materials). The structure of **2a** was unambiguously confirmed by single-crystal X-ray analysis [43], as shown in Figure 2.

## 3. Conclusions

In summary, an amide-assisted rearrangement reaction of hydroxybenzimidoyl chloride has been developed for the preparation of 1,3-diphenylurea derivatives. This highly effective reaction proceeds well to afford 1,3-diphenylurea derivatives without metal catalysts under mild conditions and shows good functional-group tolerance. A proposed reaction mechanism has been presented, suggesting that the reaction went through a novel rearrangement process. We believe that the findings of this study promote the rapid synthesis of novel diphenylurea compounds exhibiting crucial biological activity.

## Figures and Tables

**Table 1:** Optimization of experimental conditions *^a,c^*.

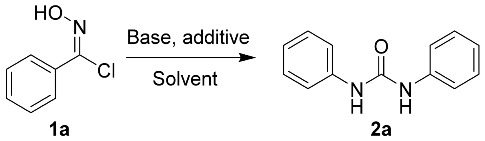						
Entry	Base	Solvent	Additive	Time (h)	*t* [°C] *^b^*	Yield (%)
1	Cs_2_CO_3_	DMF	Benzamide	5	rt *^b^*	31
2	Cs_2_CO_3_	MeOH	Benzamide	5	rt *^b^*	nr
3	Cs_2_CO_3_	EtOH	Benzamide	5	rt *^b^*	nr
4	Cs_2_CO_3_	DCM	Benzamide	5	rt *^b^*	nr
5	Cs_2_CO_3_	Toluene	Benzamide	5	rt *^b^*	8
6	Cs_2_CO_3_	THF	Benzamide	5	rt *^b^*	nr
7	Cs_2_CO_3_	Dioxane	Benzamide	5	rt *^b^*	11
8	Cs_2_CO_3_	DMSO	Benzamide	5	rt*^b^*	42
9	Cs_2_CO_3_	Acetone	Benzamide	5	rt *^b^*	nr
10	K_2_CO_3_	DMSO	Benzamide	5	rt *^b^*	25
11	Et_3_N	DMSO	Benzamide	5	rt *^b^*	27
12	DMAP	DMSO	Benzamide	5	rt *^b^*	22
13	DBU	DMSO	Benzamide	5	rt *^b^*	19
14	tBuOK	DMSO	Benzamide	5	rt *^b^*	34
15	None	DMSO	Benzamide	5	rt *^b^*	nr
16	Cs_2_CO_3_	DMSO	Benzamide	5	30	45
17	Cs_2_CO_3_	DMSO	Benzamide	5	45	58
18	Cs_2_CO_3_	DMSO	Benzamide	5	60	61
19	Cs_2_CO_3_	DMSO	Benzamide	5	75	66
20	Cs_2_CO_3_	DMSO	Benzamide	5	90	73
21	Cs_2_CO_3_	DMSO	Benzamide	5	120	87
22	Cs_2_CO_3_	DMSO	Benzamide	5	reflux	87
23	Cs_2_CO_3_	DMSO	Benzamide	10	120	87
24	Cs_2_CO_3_	DMSO	Acetamide	5	120	71
25	Cs_2_CO_3_	DMSO	Propionamide	5	120	62
26	Cs_2_CO_3_	DMSO	Phenylacetamide	5	120	39
27	Cs_2_CO_3_	DMSO	Chlorobenzamide	5	120	trace
28	Cs_2_CO_3_	DMSO	None	10	120	nr
29	Cs_2_CO_3_	DMSO	Benzamide (0.2 mmol)	10	120	63
30	Cs_2_CO_3_	DMSO	Benzamide (0.1 mmol)	10	120	46
31	Cs_2_CO_3_	DMSO	Benzamide (0.5 mmol)	10	120	88

*^a^* Reagents and conditions: N-hydroxybenzimidoyl chloride (**1a**, 1.0 mmol), benzamide (0.4 mmol), base (2.2 mmol), solvent (20 mL). *^b^* rt = room temperature. *^c^* Isolated yield based on **1a**.

**Table 2:** Synthesis of 1,3-diphenylurea derivatives *^a,b^*.

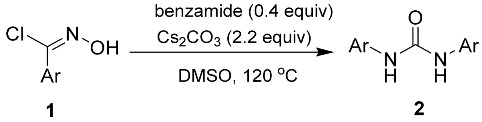
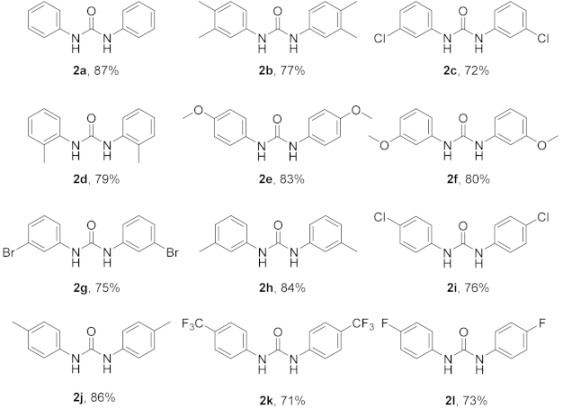

*^a^* General conditions: N-hydroxybenzimidoyl chloride (**1a**, 1.0 mmol), benzamide (0.4 mmol), Cs_2_CO_3_ (2.2 mmol), DMSO (20 mL). *^b^* Isolated yield based on **1**.

## Data Availability

The data generated during the present study are available from the corresponding author on reasonable request.

## Supplementary Materials

The following are available online, X-ray crystallography data of compounds: **2a**, Characterization data and copies of ^1^H and ^13^C NMR spectra for all new compounds.

## Sample Availability

Samples of all the compounds are available from the authors.
