# Supplementary material for: Amide-Assisted Rearrangement of Hydroxyarylformimidoyl Chloride to Diarylurea
Source: Molecules. 2021 Oct 25;26(21):6437. doi: 10.3390/molecules26216437 (PMC8587945; doi:10.3390/molecules26216437)
Supplement: Supplementary file 1 [file molecules-26-06437-s001.zip › molecules-1417041-supplementary.pdf]

Supporting Information

Amide-assisted rearrangement of hydroxyarylformimidyl chloride to diarylurea

Xizhong Song [2], Xiaoyu Liu [1], Wei Yu *[3] and Yi Jin *[1]

[1] Key Laboratory of Medicinal Chemistry for Natural Resource, Ministry of Education and Yunnan Province, School of Chemical Science and Technology, Yunnan University, Kunming, 650091, P. R. China.; eachnet99@126.com
[2] Jianxi Nafutang Pharmaceutical Co., Ltd, Zhanshu, 331200; 519672429@qq.com
[3] Pharmaceutical Department, Kunming General Hospital of Chengdu Military Command, Kunming 650118, PR China;
* Correspondence: allienyu@163.com (W. Y.); jinyi@ynu.edu.cn (Y. J.); Tel.: 86-0871-65033119

Keywords: Rearrangement, Urea, Benzamide, Hydroxybenzimidoyl chloride.

1. Materials and Methods

All chemicals and reagents were used of commercial grade and were used without no further purification. Reactions were monitored by thin layer chromatography using UV light to visualize the course of reaction. Purification of reaction products was carried out by flash chromatography on silica gel. Column chromatography was performed with 200–300 mesh silica gel. Chemical yields refer to pure isolated substances. ^{1}H spectra were obtained using a Bruker DPX-600 spectrometer. Chemical shifts were reported in ppm with TMS as the internal standard. The following abbreviations were used to designate chemical shift multiplicities: s = singlet, d = doublet, t = triplet, q = quartet, h = heptet, m = multiplet, br = broad. All coupling constants (J values) are reported in Hertz (Hz). HRMS were obtained on an Agilent LC/MSD TOF instrument by electrospray ionization (ESI). Melting points were obtained on an XT-4A micro-melting point apparatus.

2. General Procedure for preparing diphenylurea derivatives 2

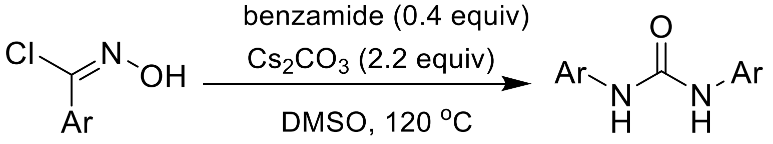

1 2

N-hydroxybenzimidoyl chloride (1 mmol), benzamide (0.4 mmol) and Cs_2CO_3 (2.2mmol) was added to 20 ml Dimethyl Sulfoxide. The mixture was stirred at 120 ℃ for 5 h and monitored by TLC. After completion, the reaction was quenched with saturated NaCl solution and extracted with EtOAc for three times. The combined organic layers were dried over Na_2SO_4. The crude materials were purified by flash column chromatography to give the desired products 2 using EtOAc/petroleum ether 1:18 as the mobile phase. The products were further identified by NMR spectroscopy and HRMS.

3. **Proposed mechanism**

Based on these experimental results, we have proposed a plausible mechanism shown in Scheme 2. Initially, amide group H-bonded with N-hydroxybenzimidoyl chloride to form an intermediate **3**, and then obtained hydrated cation intermediate **4**. Followed by dehydration, the aryl group of the oxime migrates to the nitrogen atom of hydroxylamine, giving chloroimine cation (**5**). Subsequently, the nitrile positive ion (**6**) obtained after electron transfer was condensed with water to obtain intermediate **7**. After removing hydrogen chloride from intermediate **7**, isocyanate (**8**) was obtained. Under the action of base, two molecules of isocyanate undergo a rearrangement process to form intermediate **9,** which produces intermediate **10** under the influence of water. Finally, intermediate **11** undergoes a decarboxylation process to obtain target product **2**.

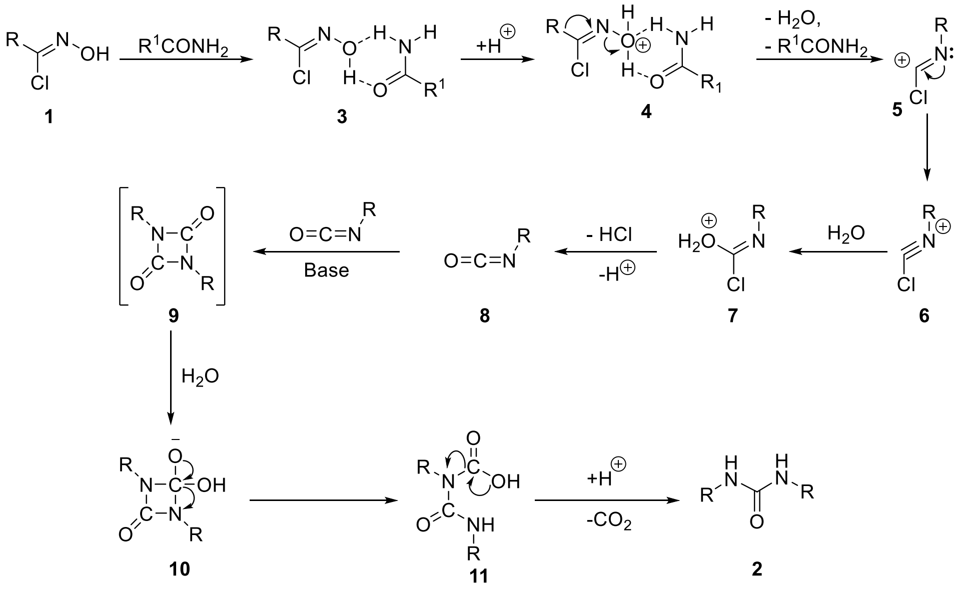

Scheme S1. Proposed mechanism

4. Spectroscopic Data of 2

4.1 Spectroscopic Data of **2a**

1,3-diphenylurea

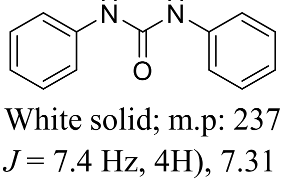

White solid; m.p: 237.7 ℃; yield: 87%; ^{1}H NMR (600 MHz, DMSO-*d*$_6$) δ 8.63 (s, 2H), 7.46 (d, *J* = 7.4 Hz, 4H), 7.31 – 7.24 (m, 4H), 6.97 (t, *J* = 7.4 Hz, 2H); ^{13}C NMR (151 MHz, DMSO-*d*$_6$) δ 153.01, 140.18, 129.19, 122.25, 118.69; HRMS (TOF ES$^-$): m/z calcd for $C_{13}H_{12}N_2O$ [M-H]$^-$, 211.0877, found, 211.0877.

4.2 Spectroscopic Data of **2b**

1,3-bis(3,4-dimethylphenyl)urea

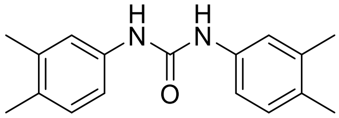

White solid; m.p: 236.4 ℃; yield: 77%; ^{1}H NMR (600 MHz, DMSO-d_6) δ 8.38 (s, 2H), 7.22 (s, 2H), 7.14 (dd, J = 8.1, 2.3 Hz, 2H), 7.01 (d, J = 8.1 Hz, 2H), 2.18 (s, 6H), 2.15 (s, 6H); ^{13}C NMR (151 MHz, DMSO-d_6) δ 153.10, 137.94, 136.74, 130.08, 129.80, 120.01, 116.21, 20.06, 19.08; HRMS (TOF ES$^+$): m/z calcd for $C_{17}H_{20}N_2O$ [M+Na]$^+$, 291.1468, found, 291.1468.

4.3 Spectroscopic Data of **2c**

1,3-bis(3-chlorophenyl)urea

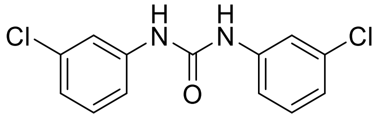

White solid; m.p: 244.8 ℃; yield: 72%; ^{1}H NMR (600 MHz, DMSO-d_6) δ 8.95 (s, 2H), 7.71 (s, 2H), 7.33 – 7.25 (m, 4H), 7.04 (d, J = 7.4 Hz, 2H); ^{13}C NMR (151 MHz, DMSO-d_6) δ 152.74, 141.46, 133.69, 130.84, 122.20, 118.31, 117.34; HRMS (TOF ES$^-$): m/z calcd for $C_{13}H_{10}N_2OCl_2$ [M-H]$^-$, 279.0097, found, 279.0161.

4.4 Spectroscopic Data of **2d**

1-(m-tolyl)-3-(o-tolyl)urea

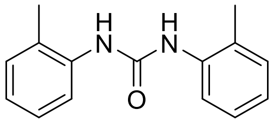

White solid; m.p: 238.5 ℃; yield: 79%; ^{1}H NMR (600 MHz, DMSO-d_6) δ 8.02 (s, 2H), 7.88 (dd, J = 7.9, 1.3 Hz, 2H), 7.00 – 6.94 (m, 4H), 6.90 (td, J = 7.3, 6.7, 1.2 Hz, 2H), 2.28 (s, 6H); ^{13}C NMR (151 MHz, DMSO-d_6) δ 146.06, 128.49, 123.72, 119.71, 118.43, 115.77, 113.01, 9.55. HRMS (TOF ES$^+$): m/z calcd for $C_{15}H_{16}N_2O$ [M+H]$^+$, 241.1335, found, 241.1335.

4.5 Spectroscopic Data of **2e**

1,3-bis(4-methoxyphenyl)urea

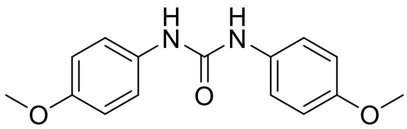

White solid; m.p: 232.2 ℃; yield: 83%; ^{1}H NMR (600 MHz, DMSO-d_6) δ 6.66 (d, J = 8.8 Hz, 4H), 6.55 (dd, J = 8.8, 2.3 Hz, 4H), 3.63 (s, 6H); ^{13}C NMR (151 MHz, DMSO-d_6) δ 156.30, 152.14, 133.42, 120.88, 115.02, 55.47. HRMS (TOF ES$^+$): m/z calcd for $C_{15}H_{16}N_2O_3$ [M+H]$^+$, 273.1234, found, 273.1236.

4.6 Spectroscopic Data of **2f**

1,3-bis(3-methoxyphenyl)urea

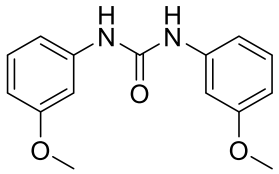

White solid; m.p: 172.9 ℃; yield: 80%; ^{1}H NMR (600 MHz, DMSO-d_6) δ 6.92 (tt, J = 8.0, 2.1 Hz, 2H), 6.21 – 6.17 (m, 4H), 6.13 – 6.09 (m, 2H), 3.66 (s, 6H); ^{13}C NMR (151 MHz, DMSO-d_6) δ 161.12, 152.92, 140.88, 130.43, 113.53, 106.11, 106.05, 55.47. HRMS (TOF ES$^+$): m/z calcd for $C_{15}H_{16}N_2O_3$ [M+H]$^+$, 273.1234, found, 273.1234.

4.7 Spectroscopic Data of **2g**

1,3-bis(3-bromophenyl)urea

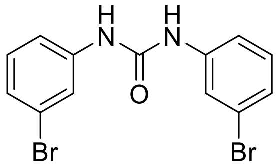

White solid; m.p: 264.3 °C; yield: 75%; ^{1}H NMR (600 MHz, DMSO-d6) δ 11.47 (s, 2H), 8.11 (s, 1H), 8.04 (d, J = 1.5 Hz, 1H), 7.94 (d, J = 7.8 Hz, 1H), 7.90 (d, J = 7.7 Hz, 1H), 7.88 – 7.77 (m, 2H), 7.53 – 7.45 (m, 2H); ^{13}C NMR (151 MHz, DMSO-d^6) δ 151.80, 139.63, 132.22, 124.90, 123.91, 120.19, 119.20. HRMS (TOF ES$^+$): m/z calcd for $C_{13}H_{10}N_2OBr_2$ [M+H]$^+$, 370.9212, found, 370.9212.

4.8 Spectroscopic Data of **2h**

1,3-di-m-tolylurea

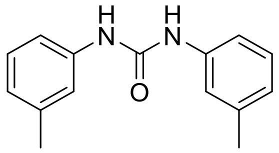

White solid; m.p: 220.7 °C; yield: 84%; ^{1}H NMR (600 MHz, DMSO-d_6) δ 8.54 (s, 2H), 7.32 (s, 2H), 7.23 (d, J = 8.1 Hz, 2H), 7.16 (t, J = 7.8 Hz, 2H), 6.79 (d, J = 7.9 Hz, 2H), 2.28 (s, 6H); ^{13}C NMR (151 MHz, DMSO-d_6) δ 152.98, 140.13, 138.40, 129.06, 123.00, 119.20, 115.85, 21.68; HRMS (TOF ES$^+$): m/z calcd for $C_{15}H_{16}N_2O$ [M+Na]$^+$, 263.1155, found, 263.1156.

4.9 Spectroscopic Data of **2i**

1,3-bis(4-chlorophenyl)urea

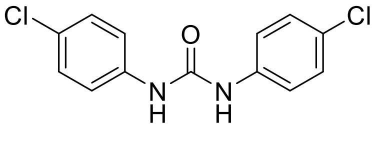

White solid; m.p: 239.6 °C; yield: 76%; ^{1}H NMR (600 MHz, DMSO-d6) δ 8.85 (s, 2H), 7.50 (d, J = 8.7 Hz, 4H), 7.33 (d, J = 8.9 Hz, 4H); ^{13}C NMR (151 MHz, DMSO-d6) δ 152.82, 139.03, 129.09, 126.00, 120.31; HRMS (TOF ES$^+$): m/z calcd for $C_{13}H_{10}Cl_2N_2O$ [M+H]$^+$, 281.0243, found, 281.0243.

4.10 Spectroscopic Data of **2j**

1,3-di-p-tolylurea

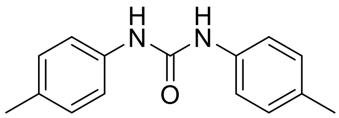

White solid; m.p: 259.9 °C; yield: 86%; ^{1}H NMR (600 MHz, DMSO-d_6) δ 12.65 (d, *J* = 19.6 Hz, 2H), 8.07 – 8.01 (m, 4H), 7.35 (d, *J* = 7.9 Hz, 4H), 2.38 (s, 6H); ^{13}C NMR (151 MHz, DMSO-d^6) δ 152.42, 137.63, 132.07, 130.10, 120.44, 20.56. HRMS (TOF ES$^+$): m/z calcd for $C_{15}H_{16}N_2O$ [M+H]$^+$, 241.1335, found, 241.1335.

4.11 Spectroscopic Data of **2k**

1,3-bis(4-(trifluoromethyl)phenyl)urea

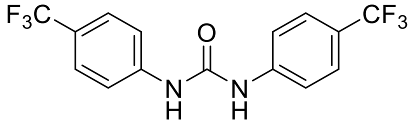

White solid; m.p: 211.1 °C; yield: 71%; ^{1}H NMR (600 MHz, DMSO-d6) δ 9.76 (s, 2H), 7.69 (d, J = 8.5 Hz, 4H), 7.64 (d, J = 8.5 Hz, 4H); ^{13}C NMR (151 MHz, DMSO-d_6) δ 143.25, 134.83, 120.46, 118.47 (*J* = 283 Hz, C-F), 117.79, 112.41. HRMS (TOF ES$^+$): m/z calcd for $C_{15}H_{10}F_6N_2O$ [M+H]$^+$, 349.0770, found, 349.0770.

4.12 Spectroscopic Data of **2l**

1,3-bis(4-fluorophenyl)urea

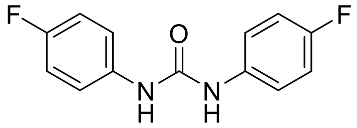

White solid; m.p: 261.2 °C; yield: 73%; ^{1}H NMR (600 MHz, DMSO-d6) δ 8.83 (s, 2H), 7.58 – 7.39 (m, 4H), 7.38 – 7.23 (m, 4H); ^{13}C NMR (151 MHz, DMSO-d_6) δ 160.19 (*J* = 259 Hz, C-F) 152.02, 135.63, 122.54, 116.07. HRMS (TOF ES$^+$): m/z calcd for $C_{15}H_{10}F_6N_2O$ [M+H]$^+$, 249.0834, found, 249.0832.

5. X-ray Structure and Data of 2a

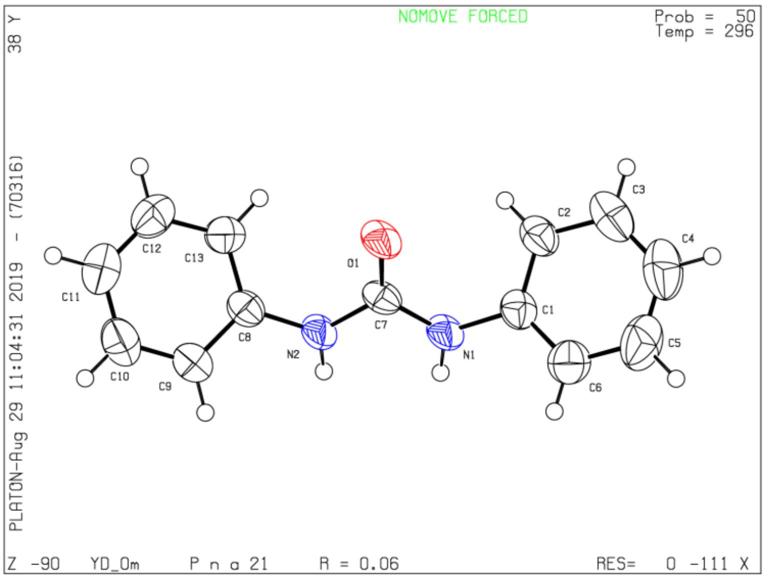

Figure S1 X-Ray crystal structure of **2a**

Datablock: YD_0m

Bond precision: C-C = 0.0081 A Wavelength=0.71073

Cell:	a=9.090(7)	b=10.562(8)	c=11.770(9)
	alpha=90	beta=90	gamma=90
Temperature:	296 K		

	Calculated	Reported
Volume	1130.0(15)	1130.0(15)
Space group	P n a 21	P n a 21
Hall group	P 2c -2n	P 2c -2n
Moiety formula	C13 H12 N2 O	?
Sum formula	C13 H12 N2 O	C13 H12 N2 O
Mr	212.25	212.25
Dx,g cm-3	1.248	1.248
Z	4	4
Mu (mm-1)	0.081	0.081
F000	448.0	448.0
F000'	448.17	
h,k,lmax	12,14,15	12,14,15
Nref	2776[1454]	2238
Tmin,Tmax	0.990,0.995	
Tmin'	0.987	

Correction method= Not given

Data completeness= 1.54/0.81 Theta(max)= 28.206

R(reflections)= 0.0606(1285) wR2(reflections)= 0.1837(2238)

S = 0.891 Npar= 145

6. NMR spectrum data of 2

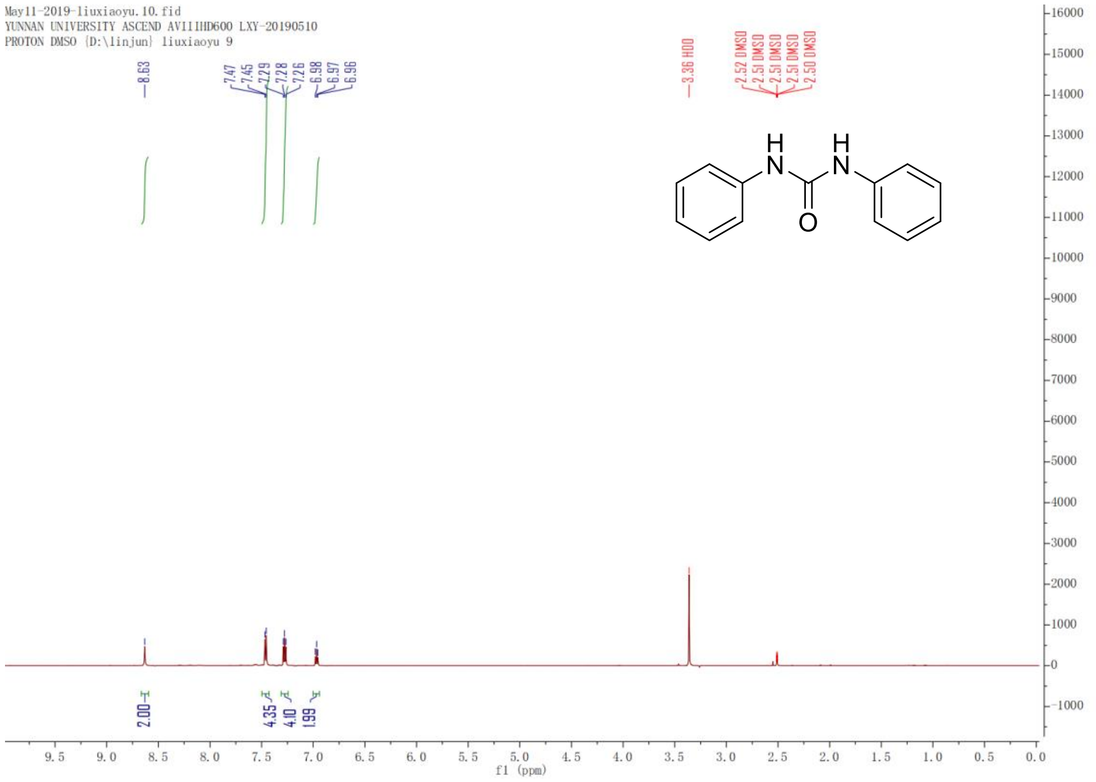

^{1}H NMR (600 MHz, DMSO) Spectra of compound **2a**

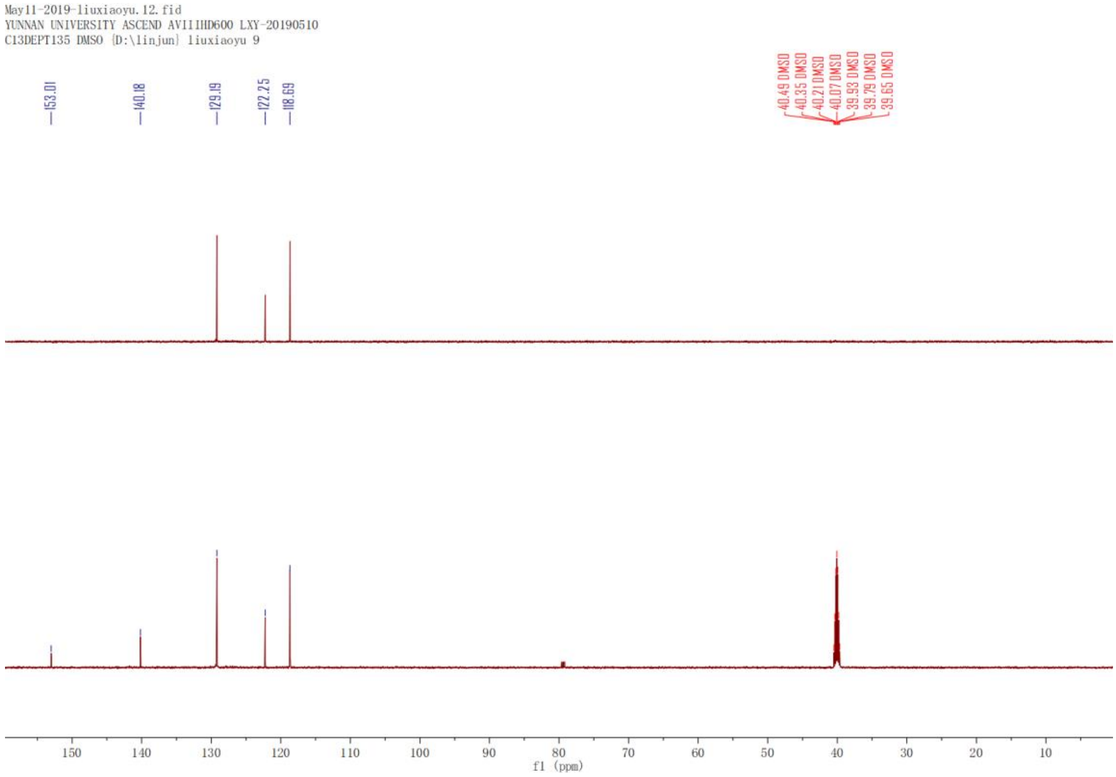

^{13}C NMR (151 MHz, DMSO) Spectra of compound **2a**

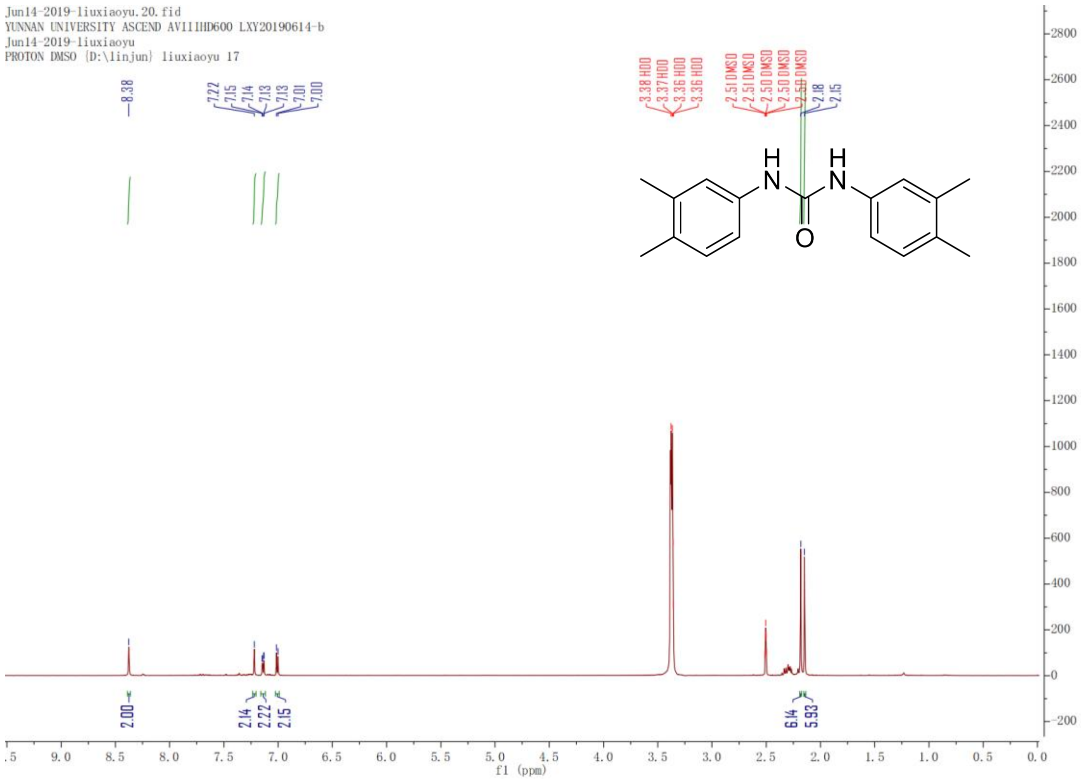

^{1}H NMR (600 MHz, DMSO) Spectra of compound **2b**

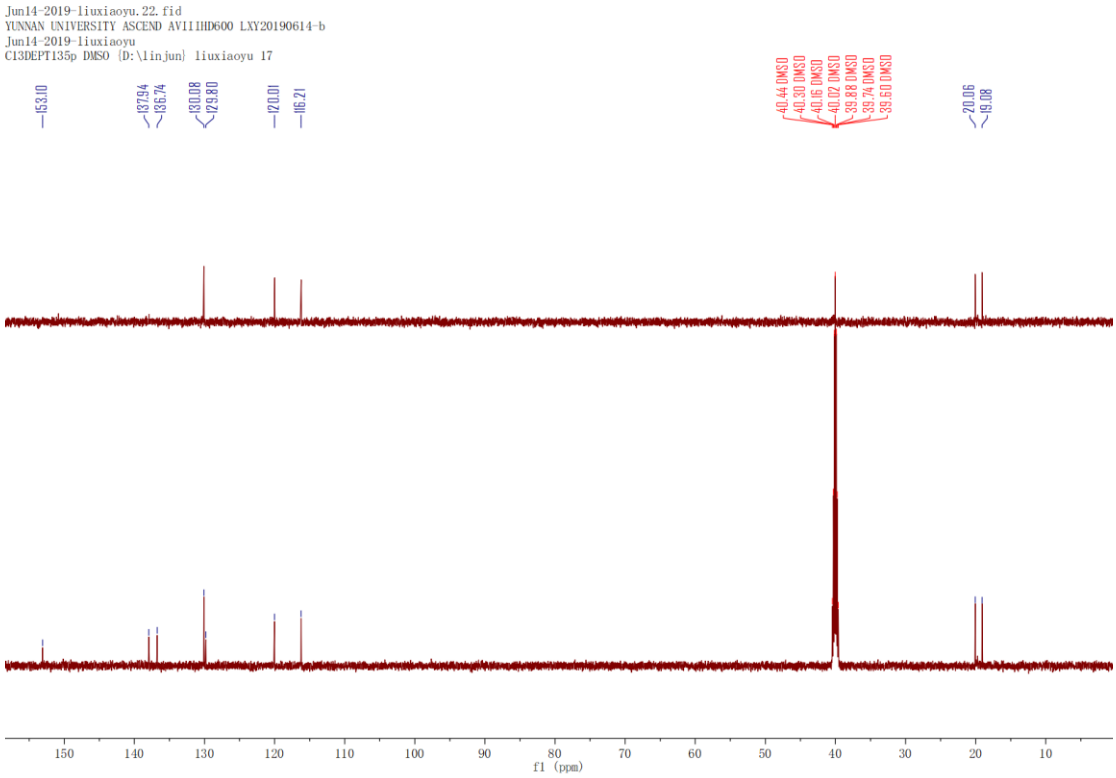

^{13}C NMR (151 MHz, DMSO) Spectra of compound **2b**

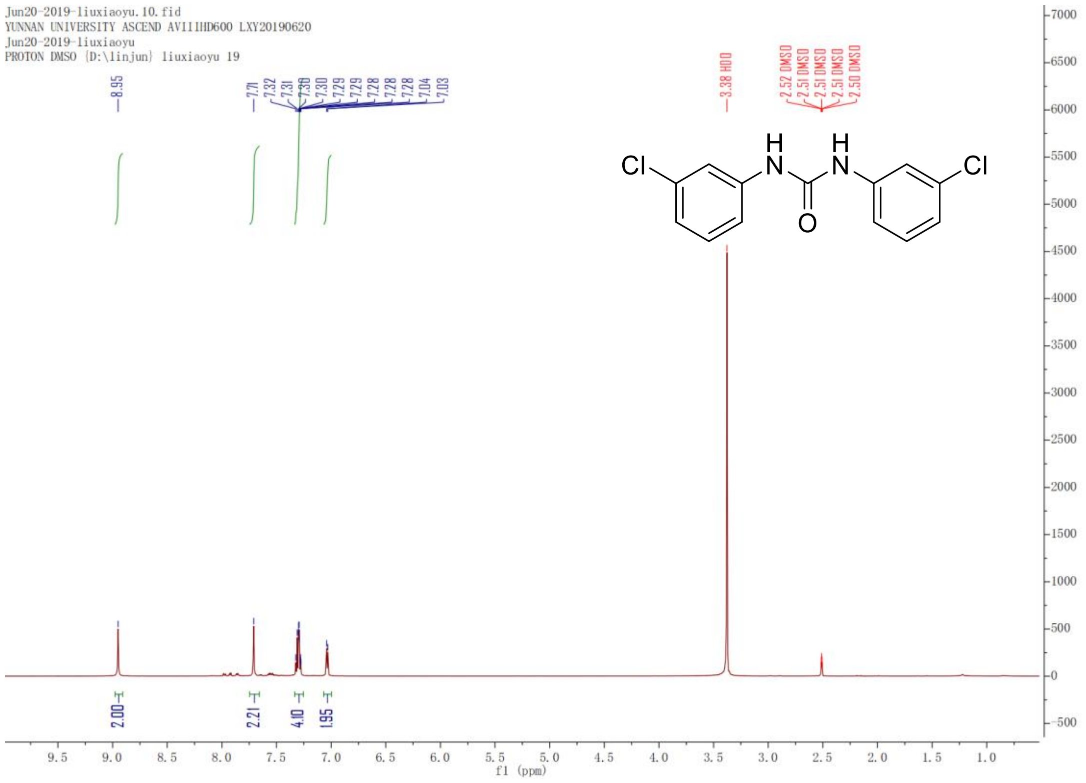

^{1}H NMR (600 MHz, DMSO) Spectra of compound **2c**

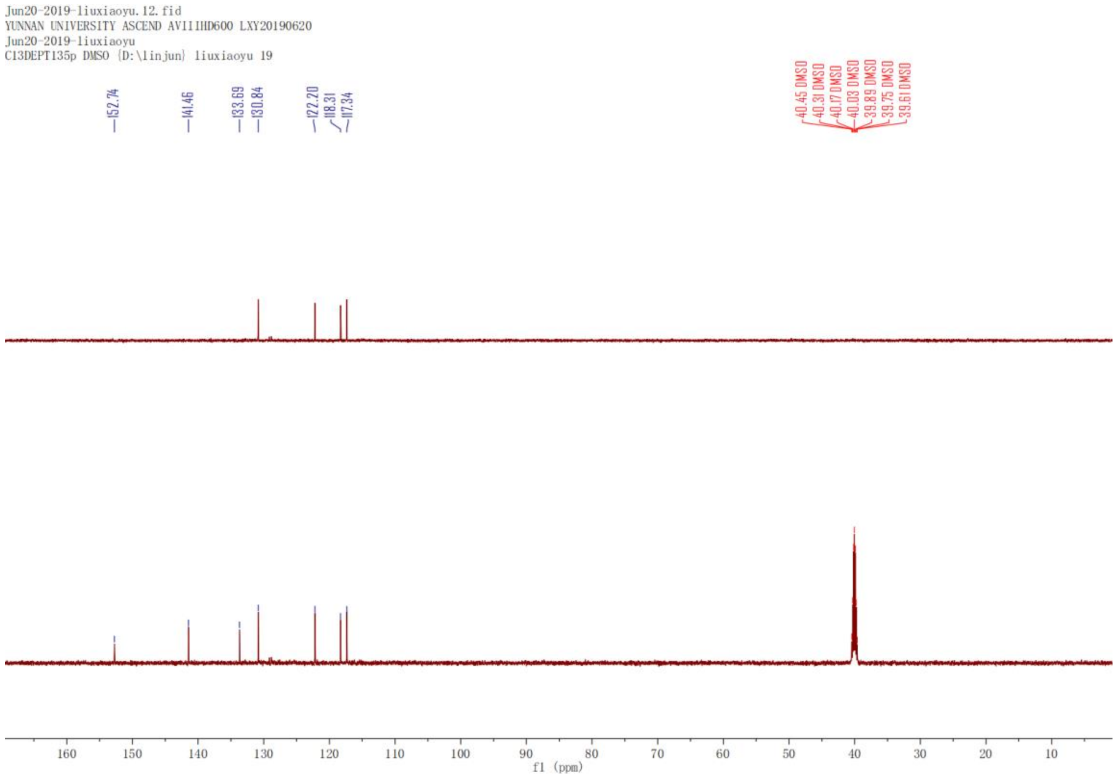

^{13}C NMR (151 MHz, DMSO) Spectra of compound **2c**

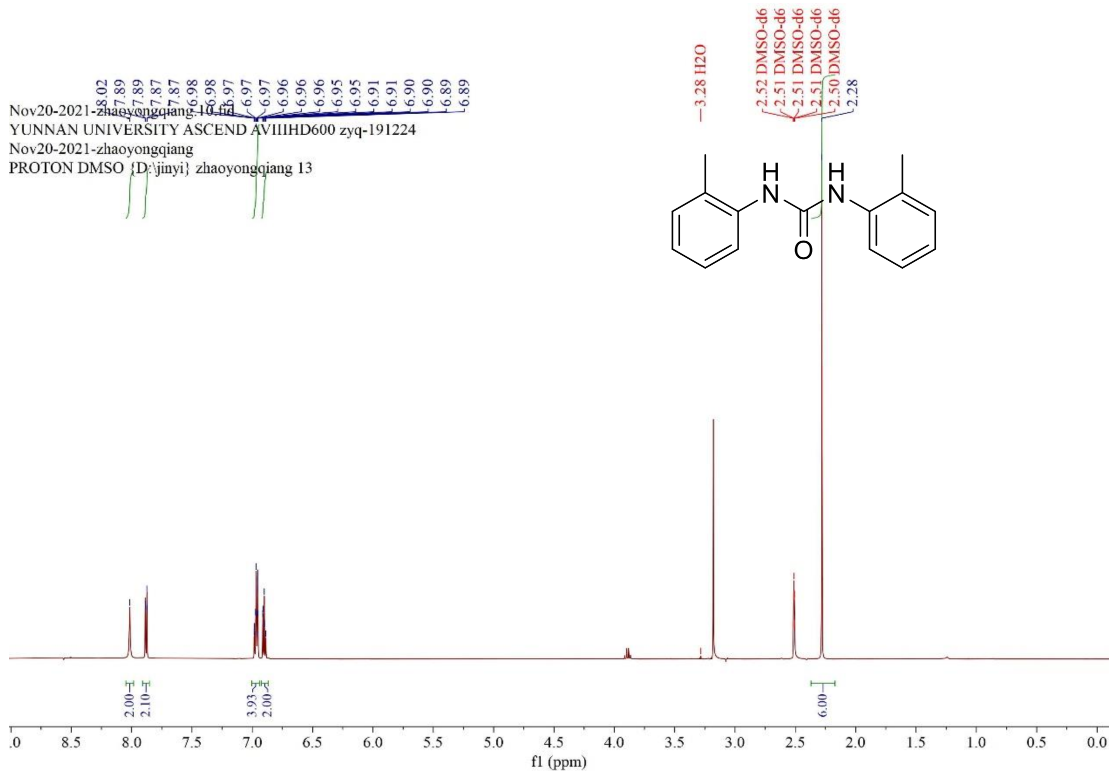

^{1}H NMR (600 MHz, DMSO) Spectra of compound **2d**

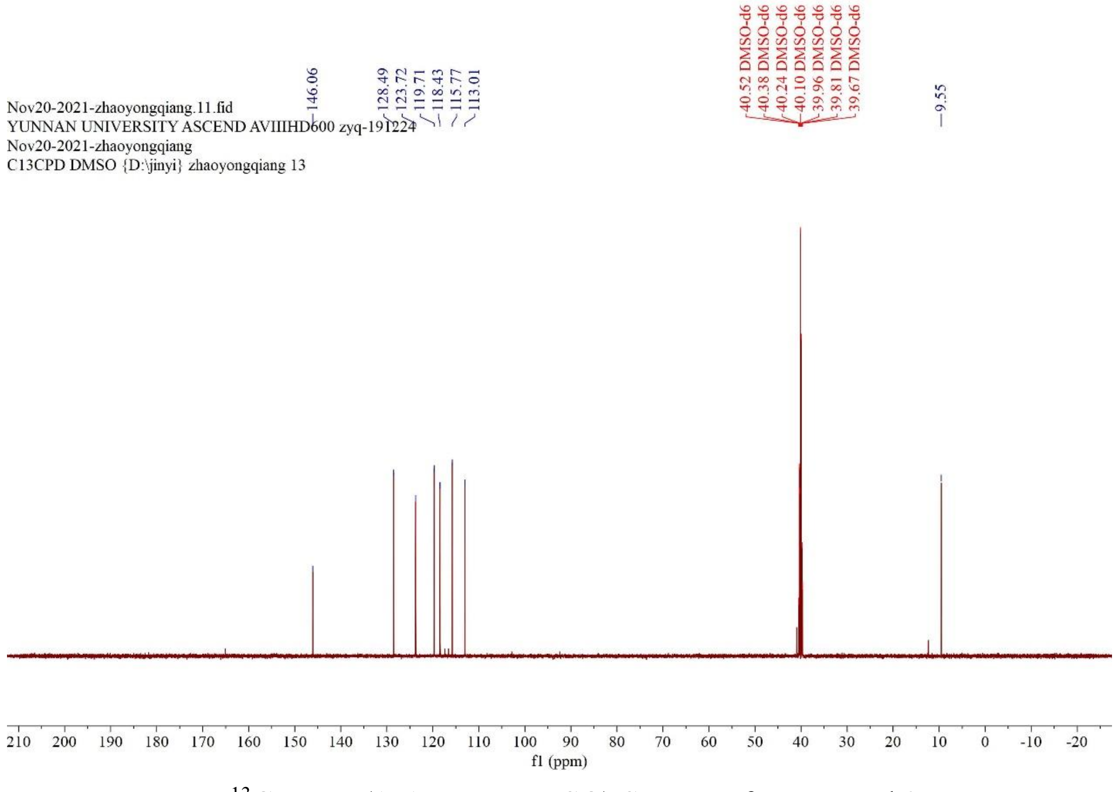

^{13}C NMR (151 MHz, DMSO) Spectra of compound **2d**

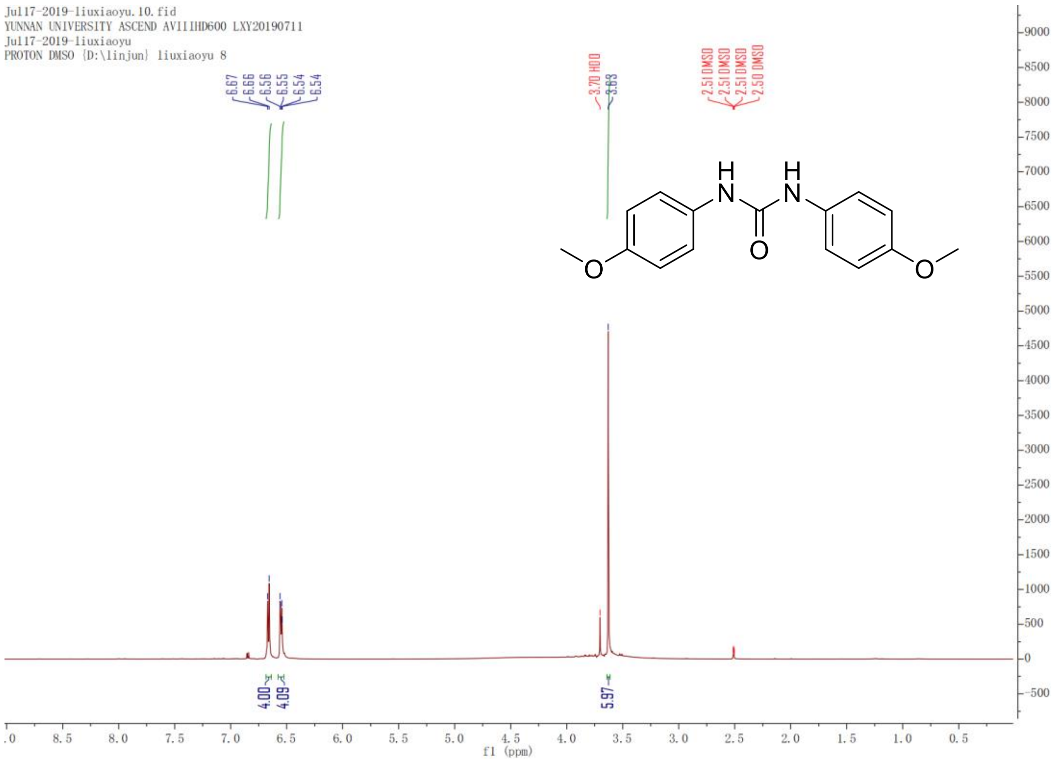

^{1}H NMR (600 MHz, DMSO) Spectra of compound **2e**

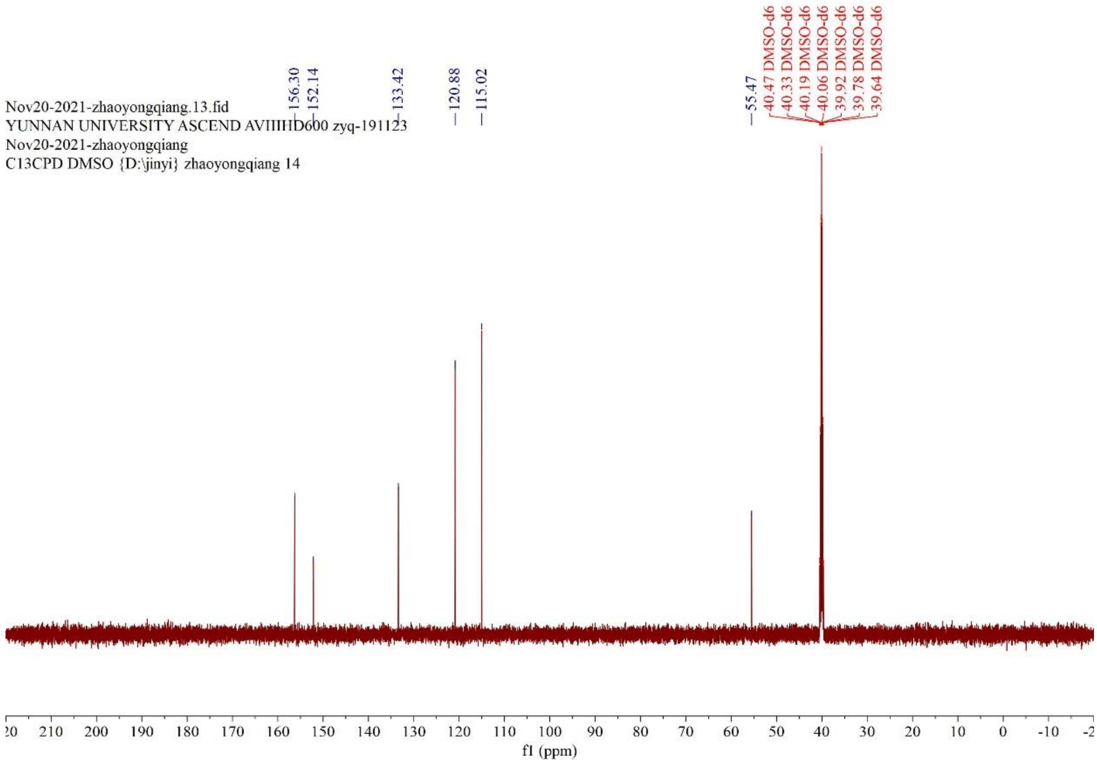

^{13}C NMR (151 MHz, DMSO) Spectra of compound **2e**

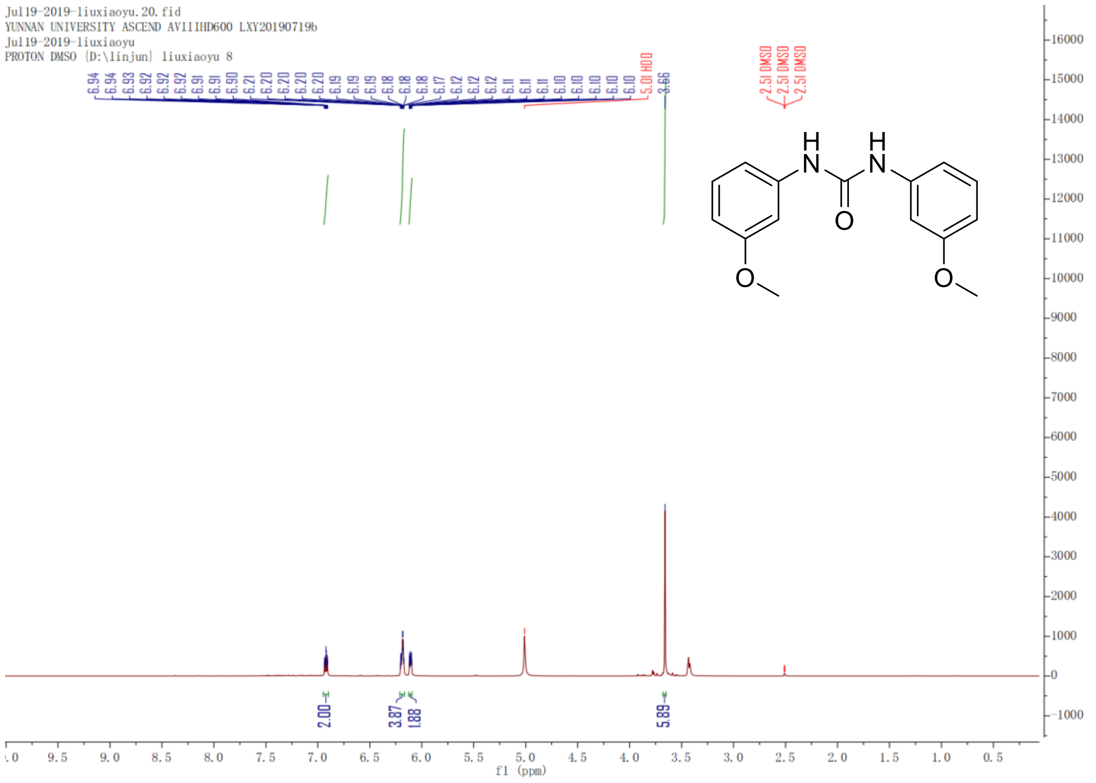

^{1}H NMR (600 MHz, DMSO) Spectra of compound **2f**

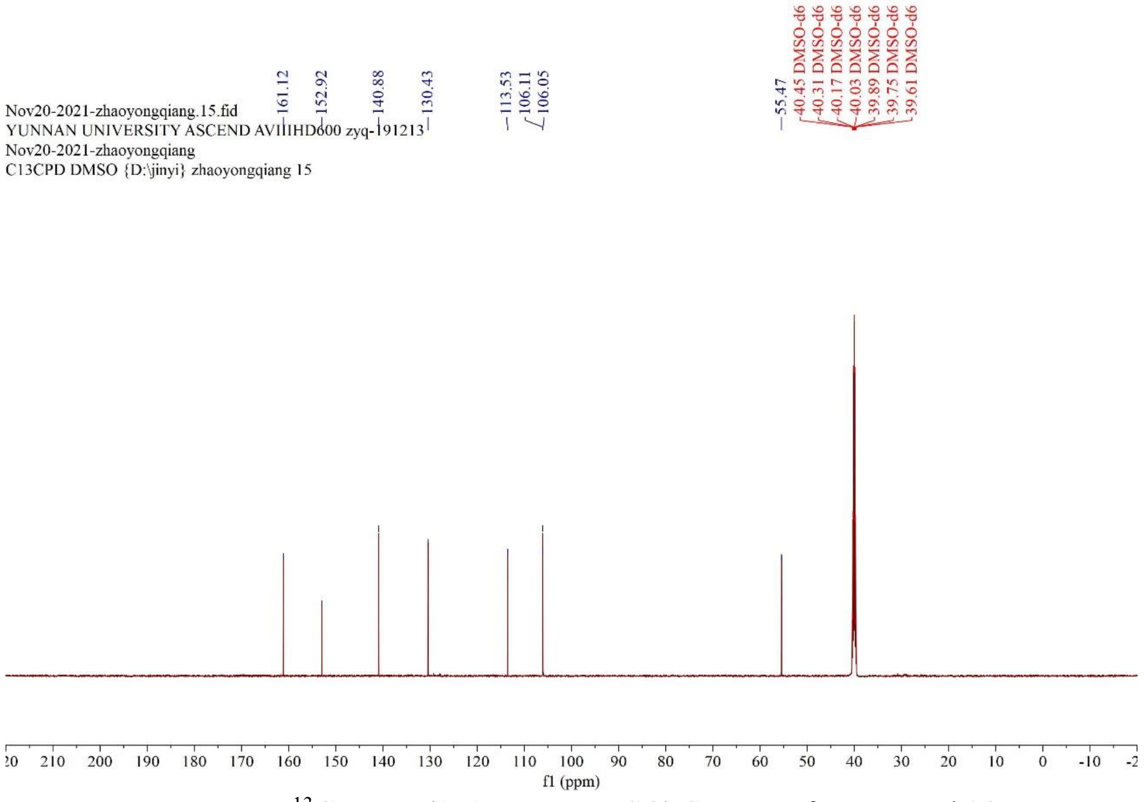

^{13}C NMR (151 MHz, DMSO) Spectra of compound **2f**

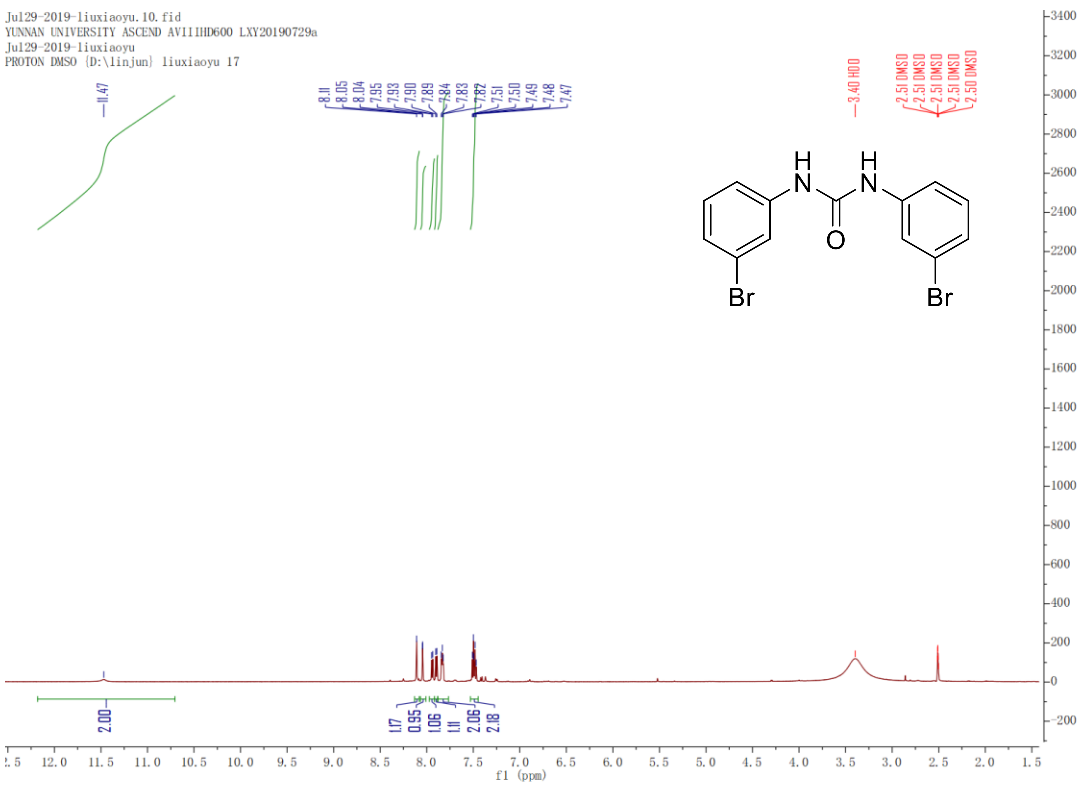

^{1}H NMR (600 MHz, DMSO) Spectra of compound **2g**

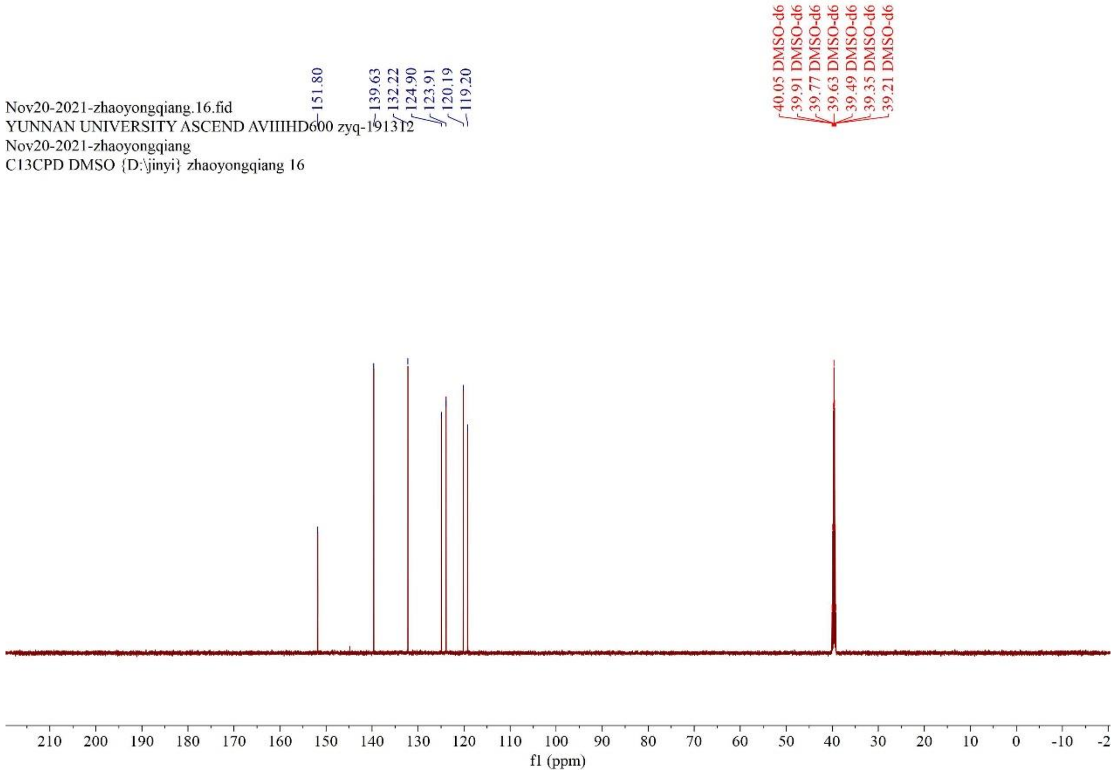

^{13}C NMR (151 MHz, DMSO) Spectra of compound **2g**

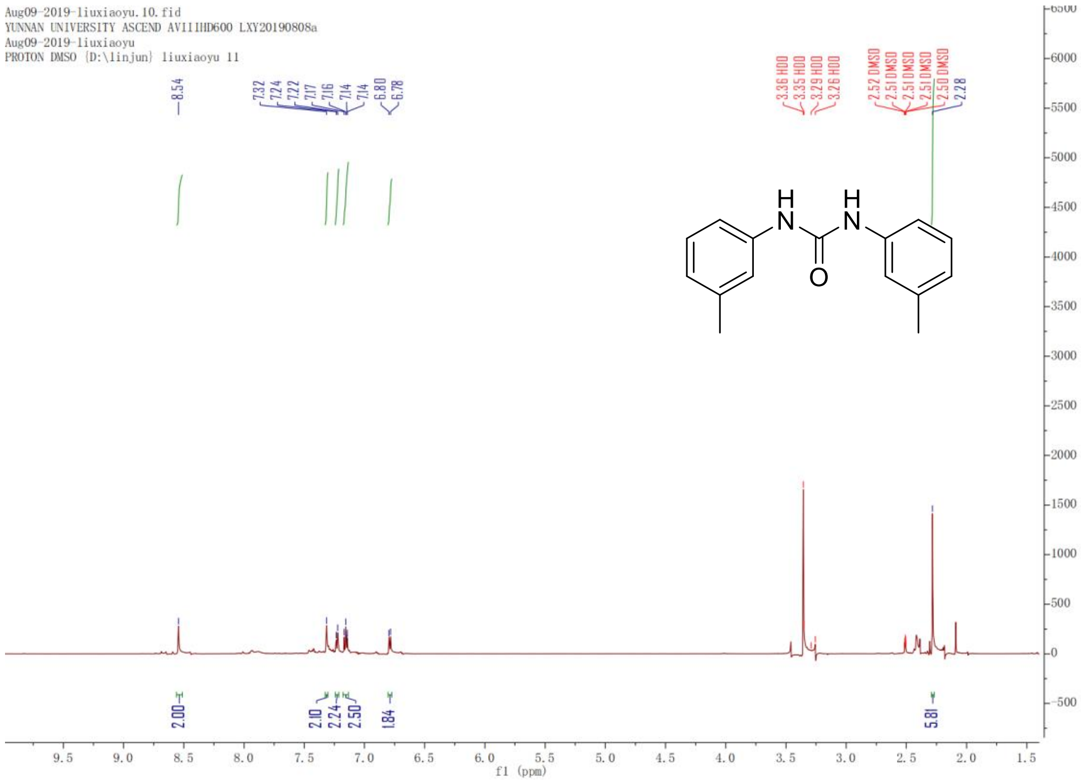

^{1}H NMR (600 MHz, DMSO) Spectra of compound **2h**

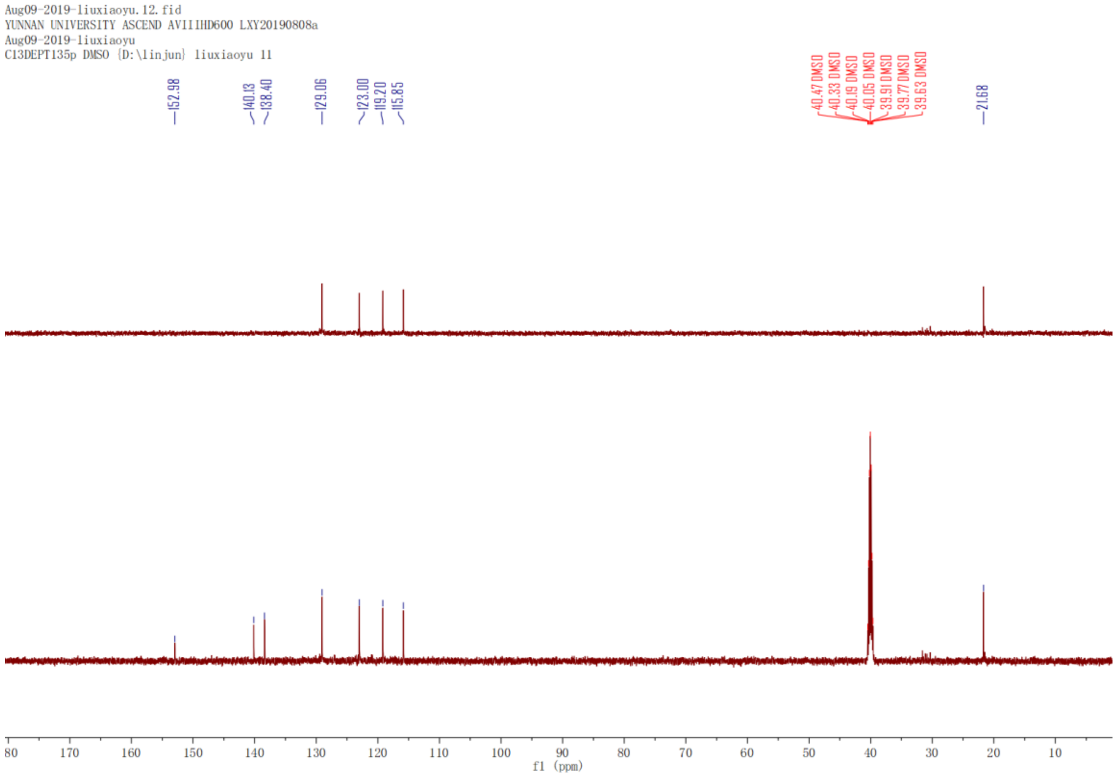

^{13}C NMR (151 MHz, DMSO) Spectra of compound **2h**

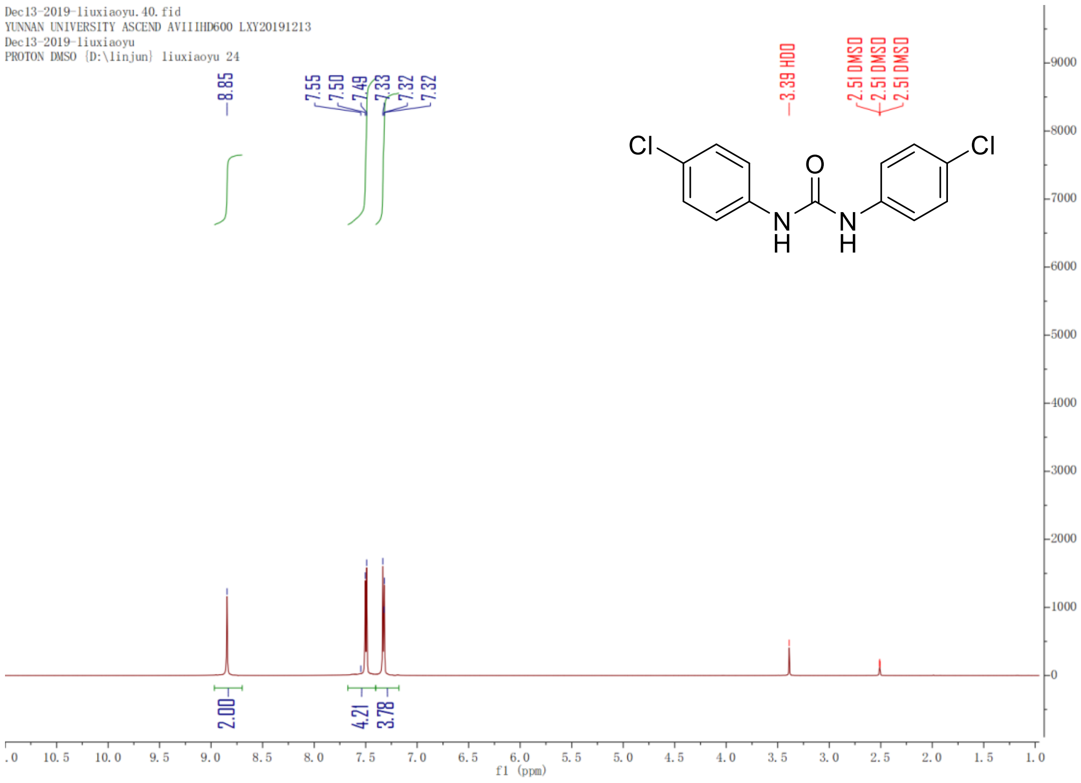

^{1}H NMR (600 MHz, DMSO) Spectra of compound **2i**

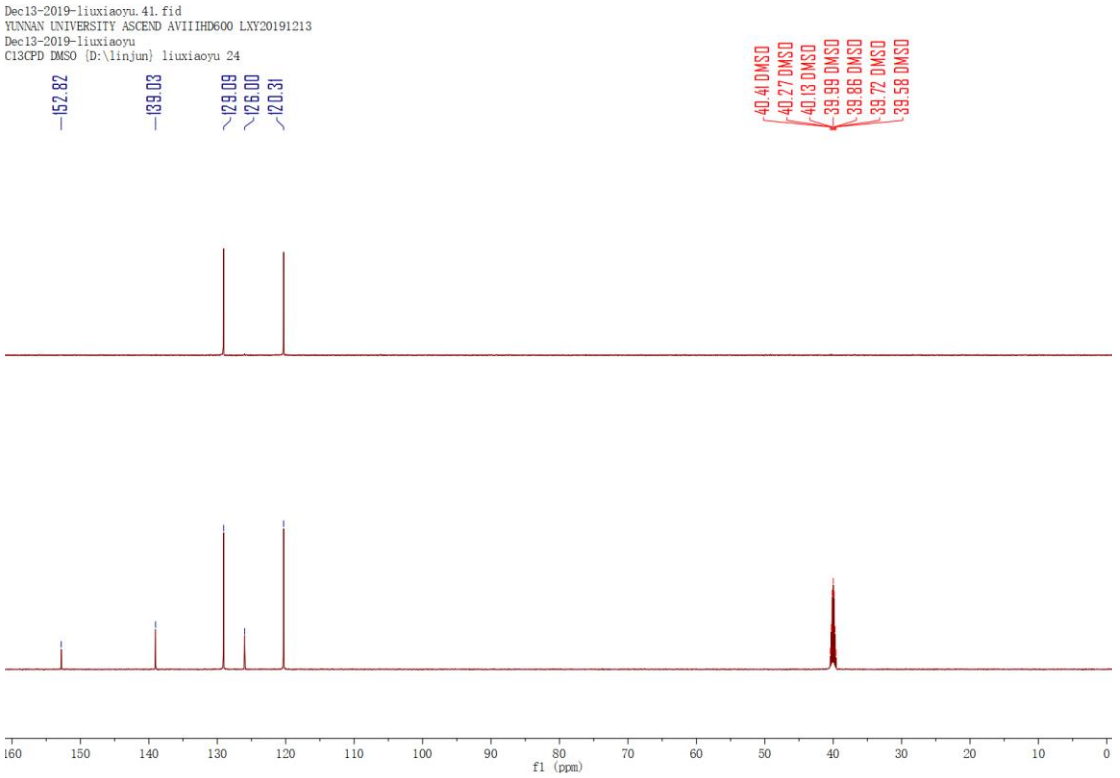

^{13}C NMR (151 MHz, DMSO) Spectra of compound **2i**

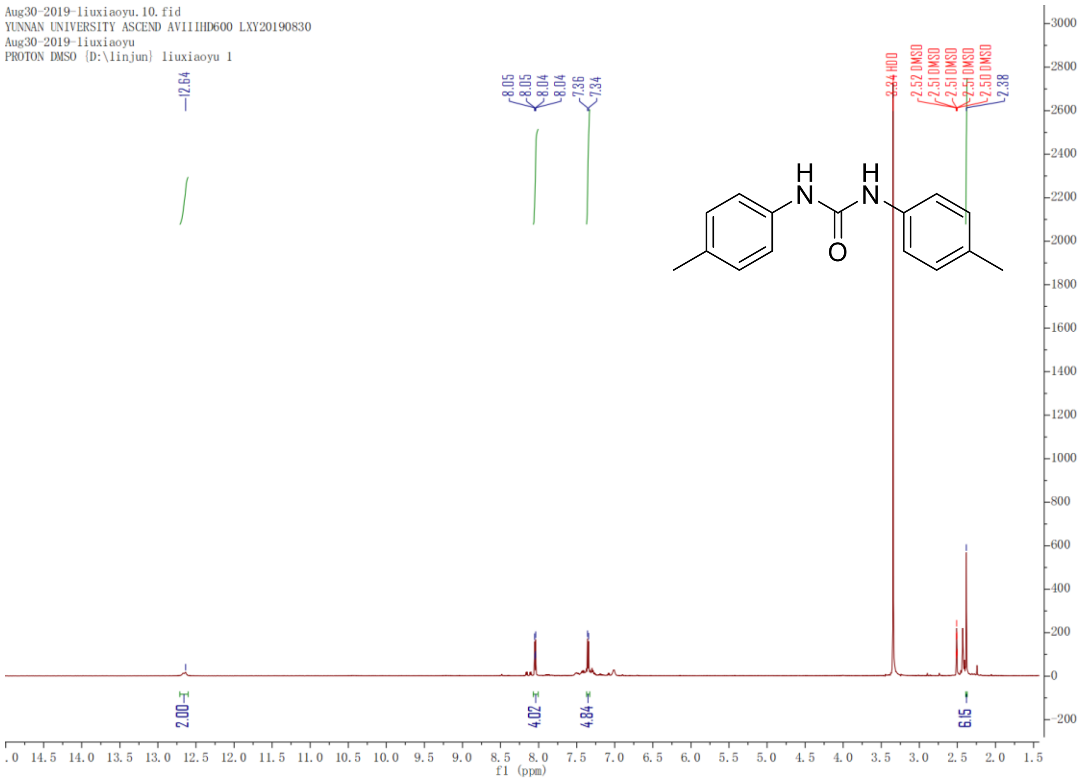

^{1}H NMR (600 MHz, DMSO) Spectra of compound **2j**

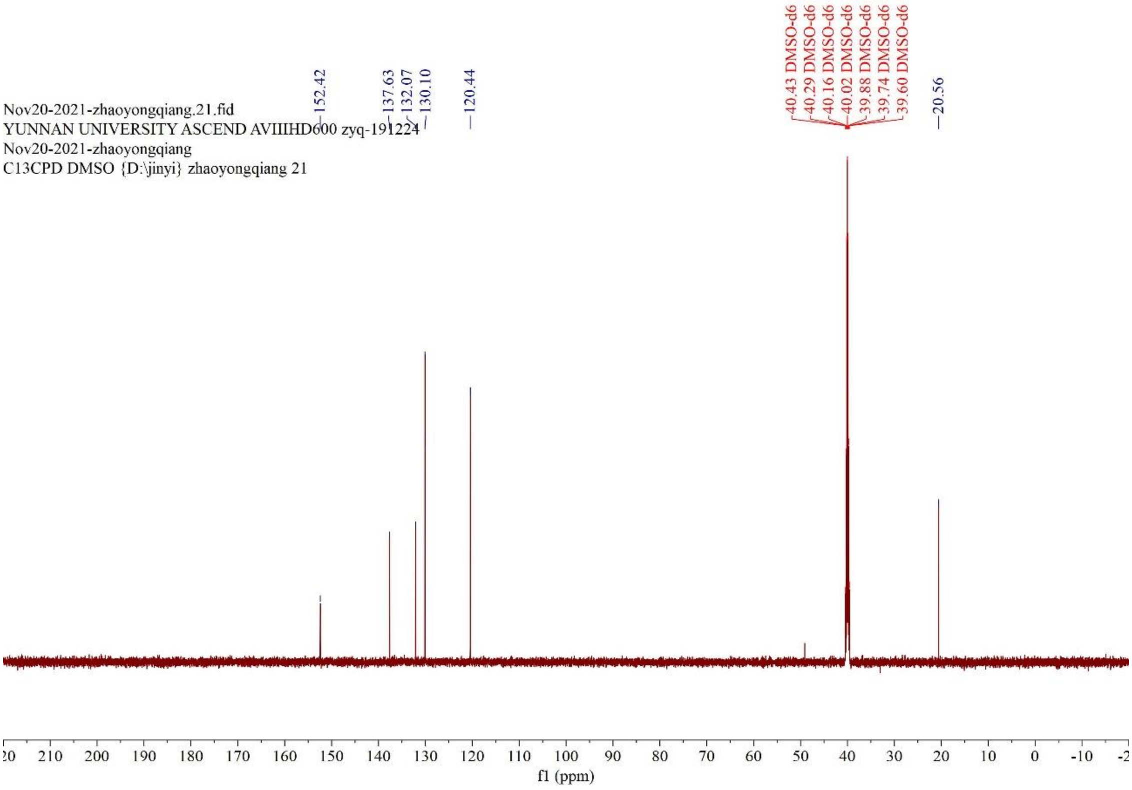

^{13}C NMR (151 MHz, DMSO) Spectra of compound **2j**

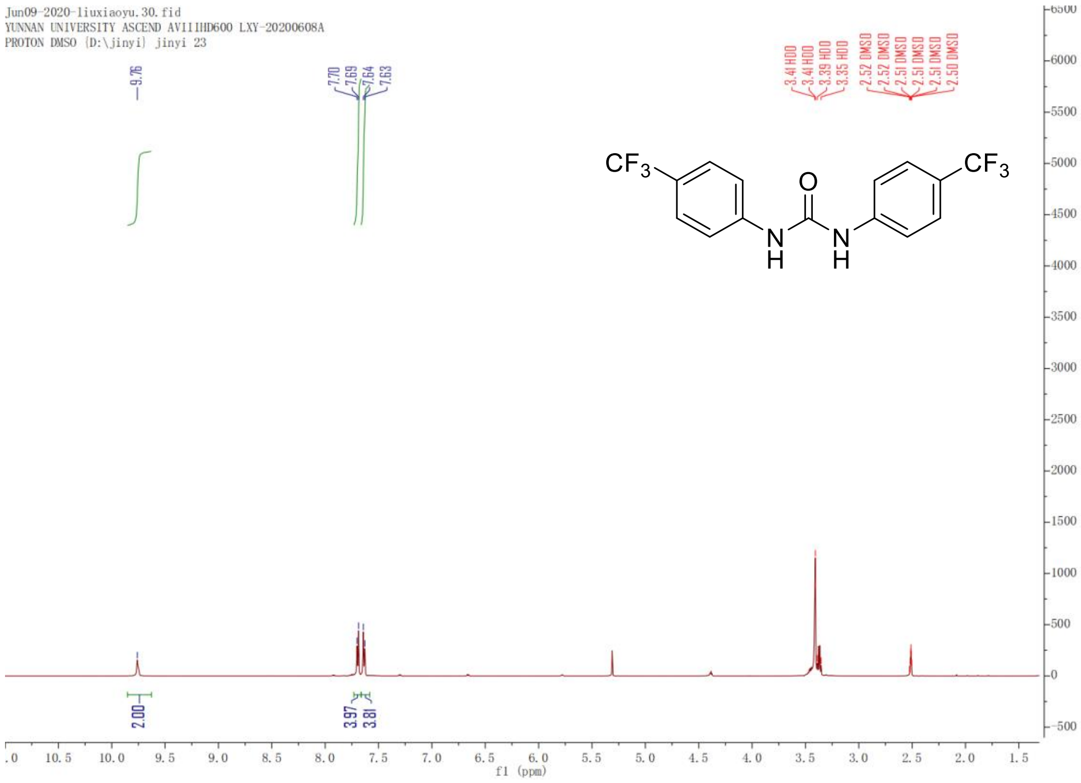

^{1}H NMR (600 MHz, DMSO) Spectra of compound **2k**

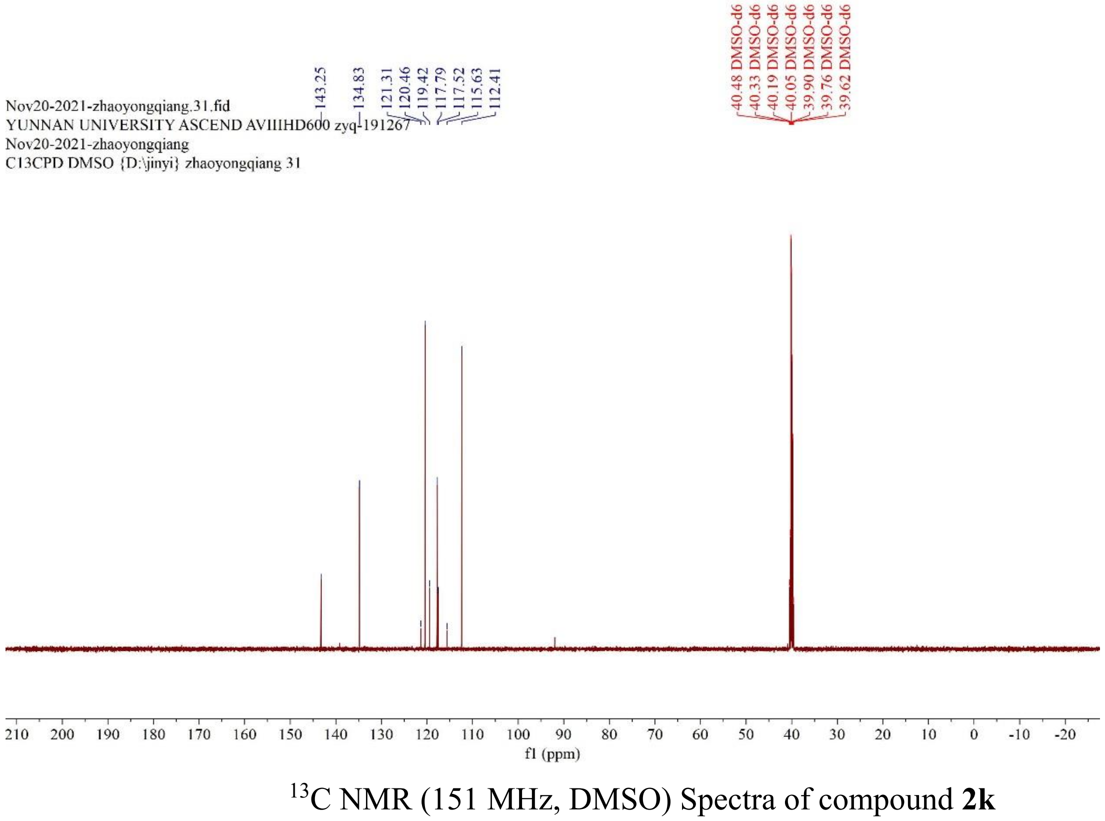

^{13}C NMR (151 MHz, DMSO) Spectra of compound **2k**

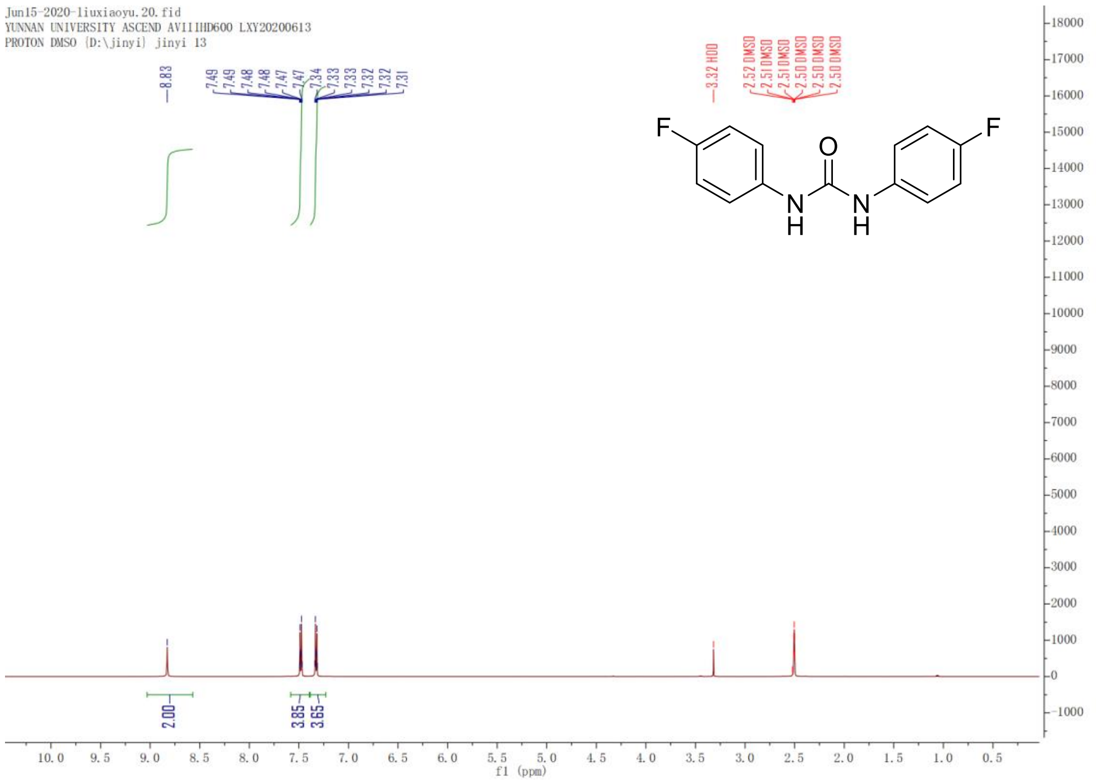

^{1}H NMR (600 MHz, DMSO) Spectra of compound **2l**

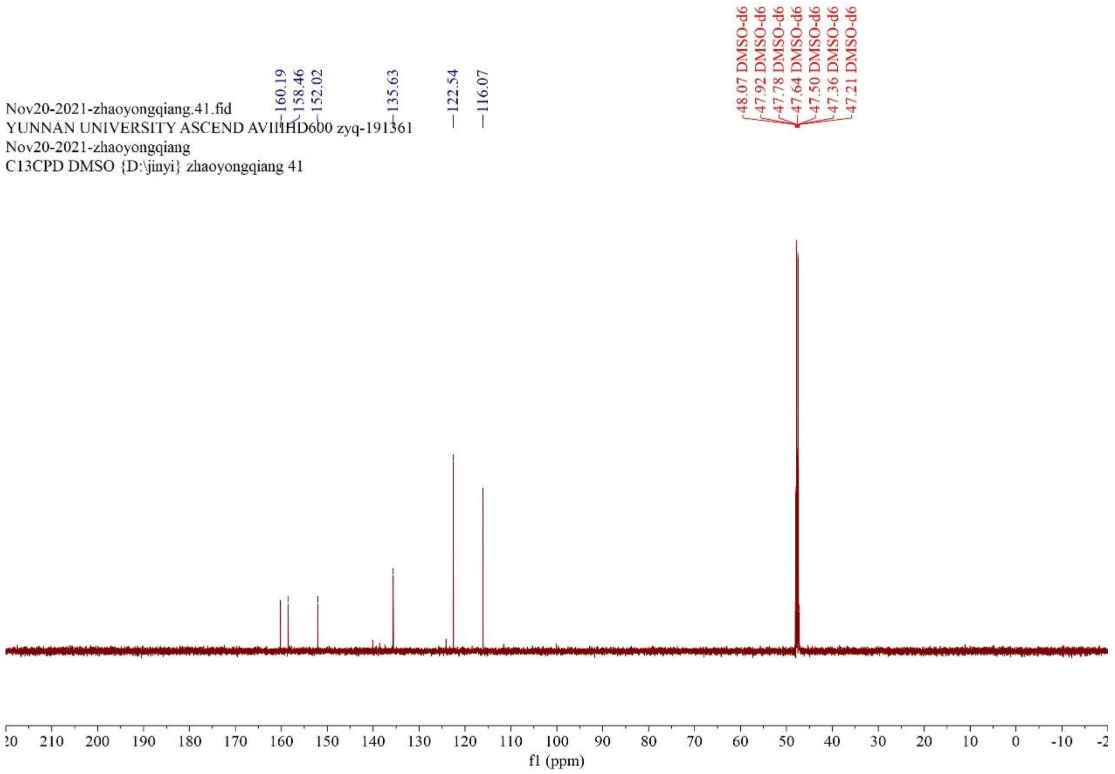

^{13}C NMR (151 MHz, DMSO) Spectra of compound **2l**
